# Bioaccumulation of Heavy Metals in Some Tissues of Fish in Lake Geriyo, Adamawa State, Nigeria

**DOI:** 10.1155/2018/1854892

**Published:** 2018-05-15

**Authors:** A. A. Bawuro, R. B. Voegborlo, A. A. Adimado

**Affiliations:** ^1^Department of Chemistry, Faculty of Physical Sciences, KNUST, Kumasi, Ghana; ^2^Department of Chemistry, Federal College of Education, Yola, Jimeta, Nigeria

## Abstract

Bioaccumulation of heavy metals (Zn, Pb, Cd, and Cu) was determined in the liver, gills, and flesh from benthic and pelagic fish species collected from Lake Geriyo covering two seasons. The levels of the heavy metals varied significantly among fish species and organs. Flesh possessed the lowest concentration of all the metals. Liver was the target organ for Zn, Cu, and Pb accumulations. Cd however exhibited higher concentration in the gills. Fish species showed interspecific variation of metals. These differences were discussed for the contribution of potential factors that affected metals uptake like age, geographical distribution, and species-specific factors. The concentration of metals in fish flesh was accepted by the international legislation limits for Cu, Zn, and Cd; however, Pb transcend in* Clarias and Tilapia* during wet season and* Heterotis* in both seasons, hence unsafe for human consumption and a threat to public health. These levels might be due to anthropogenic inputs as there is no industrial activity around the lake.

## 1. Introduction

The consumption of fish worldwide has increased speedily in recent years particularly with the awareness of its nutritional and therapeutic benefits. In addition to being important source of protein, fish are enriched with essential minerals, vitamins, and unsaturated fatty acids [1]. The American Heart Association recommended consumption of fish at least twice per week in order to reach the daily intake of omega-3 fatty acids [2]. However, fish normally accumulate heavy metals from food, water, and sediments [3, 4] and this is a good indicator of heavy metals contamination in water [5].

The presence of toxic heavy metals in fish can invalidate their beneficial effects. Several unfavorable effects of heavy metals to human health have been known for long time [6]. This includes serious threats like renal failure, liver damage, cardiovascular diseases, and even death [7, 8]. Thus, many local and international monitoring programs have been established in order to assess the quality of fish for human consumption and to monitor the health of the aquatic ecosystem [9].

According to the literatures, metal bioaccumulation by fish and subsequent distribution in organs is greatly interspecific. In addition, many factors can influence metal uptake like sex, age, size, reproductive cycle, swimming pattern, feeding behavior, and geographical location [4, 10].

The Lake Geriyo reservoir receives copious amounts of wastes as runoff due to agricultural activities and atmospheric emissions. The urban wastes management and garbage disposal system practices in the area are very poor whereby part of lake environment is used as general dumpsite of the urban. The lakes provide substantial level of local economic activities involving fish production and irrigation. They have a potential irrigable land area of about 1500 hectares with 1200 hectares already developed for the cultivation of rice and vegetables for food security. The Lake Geriyo irrigation project scheme provides vital employment opportunities for the teaming youth within Yola and Jimeta Metropolis. Information concerning the impact of pollution load on the lake matrices is scanty or not available. Therefore, this study is necessary considering the health risk of heavy metals in plants, animals, and people within the environment.

## 2. Materials and Methods

### 2.1. The Study Area

**Figure 1:**
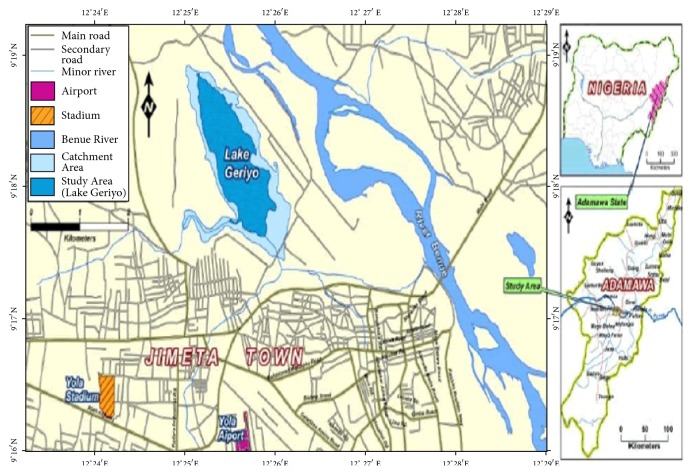
Map of Lake Geriyo.

**Figure 2:**
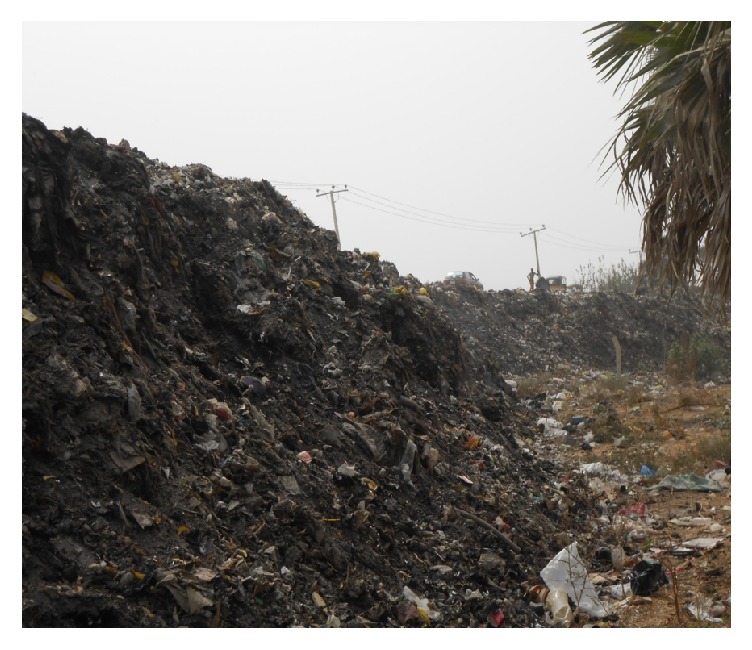
Map of the study area showing the waste dump site.

Lake Geriyo is situated in Jimeta/Yola, Adamawa State capital of Nigeria, and located on latitude 09°1811N and longitude 12°2536E (Figure 1). Lake Geriyo Occupies natural depression near the upper River Benue in the north eastern Nigeria. The lake is flooded by the river during the raining season such that it receives influx of waters which include pollution load originated from River Benue and from the urban waste dumpsite at the surrounding of the lake (Figure 2). It is a shallow water body of about 250 hectares in size with a mean depth of about 3 meters. Aquatic vegetation on the lake consists of mass of floating weeds such as water spinach, water hyacinth, water Lilly, and water lettuce which move around the lake surface due to the prevailing wind [11]. The area is in the Sahel region of Northern Nigeria generally semiarid with low rainfall, low humidity, and high temperature. The area experiences two distinctive wet and dry seasons. The wet season starts from May to October, while the dry season commences from November to April, mean daily temperature fluctuates with season from 25°C to 45°C, and mean annual rainfall received is in the range of 150–1000 m. Cold and dusty weather from December to January is followed by intense heat of March to April. The climate is characterized by high evapotranspiration especially during dry season [12].

### 2.2. Fish Sampling

Fifteen commercial fish species were caught using Malian nets set overnight with the help of the local fishermen at the lake basin in the two seasons. The collected fish species were* Clarias anguillaris, Heterotis niloticus,* and* Tilapia zilli.* These fish species represent different biotopes (Table 1) that were immediately preserved on ice in an ice chest and transferred to the laboratory where they were classified, weighed, with total length recorded, and kept frozen at −20°C until further analysis. Identification of fish to species was done by a specialist from Department of Fisheries, Modibbo Adama University of Science and Technology (MAUTECH), Yola, Nigeria.

### 2.3. Determination of Metal Concentrations

The fish samples were rinsed with distilled water and scales of* Heterotis niloticus* and* Tilapia zilli* were removed. The fishes were dissected into separate organs flesh, liver, and gills using stainless steel instruments [5, 17] and digested by the method described by Voegborlo et al. [5].

In the procedure 1 g of the samples was digested with perchloric acid and nitric acid ratio (1 : 1) HNO_3_-HCLO_4_, followed by sulphuric acid, and the mixture was heated at 200°C for 30 mins. The complete digest was then cooled down to room temperature and made up to 50 ml scale with distilled water and analysed for Cu, Zn, Pb, and Cd using Atomic Absorption Spectrophotometer (AAS model AgilentAA55) after selecting the various wavelengths at which the heavy metals were determined. An analytical blank was prepared in a similar manner. The obtained results were expressed as mg/kg wet weight.

### 2.4. Quality Assurance

All the reagents used were of analytical grade. Glass wares were soaked in 10% nitric acid for 24 hrs and rinsed with distilled water followed by 0.5% (w/v) KMNO_4_ solution and finally with distilled water. Accuracy and precision were verified by using reference material (CRM IAEA 407) provided by the International Atomic Agency (IAEA). Analytical results of the quality control samples indicated a satisfactory performance of heavy metal determination within the range of certified values 95–101% recovery for the metals studied.

### 2.5. Statistical Analysis

All analyses were performed in triplicate. Statistical data analyses of the results were performed using GRAPH PAD INSTAT AND PAST WINDOWS 2010 (computer package). The means of the replicates and evaluation of significant differences between different samples were determined using descriptive statistics and analysis of variance (ANOVA), respectively. Two-way and one-way analysis of variable (ANOVA) were used to test for significant differences in the concentrations of heavy metals in the samples along the seasons and the sites. For comparison of means, ANOVA test and post hoc Duncan test were used. Results of the test were considered significant if the calculated *P* values were ≤0.05. Pearson correlation was used to examine the relationship between the elements in fish. *T*-test was used to show the variation of data between the two seasons. Tukey pairwise comparison method was also used.

## 3. Results and Discussions

The characteristics of the fish species studied revealed that* Clarias* was carnivorous, while* Heterotis* and* Tilapia* were omnivorous or herbivorous (Table 1). There were marked variations in the concentrations of heavy metals (Cu, Zn, Pb, and Cd) in flesh, liver, and gills of the fish collected from Lake Geriyo basin (Table 2). Findings show that all fish species contained higher concentration of metals in liver and lower in flesh with few exceptions. The results of analysis of variance showed significant differences in metal concentrations in the different internal organs at (*P* < 0.05) in both seasons. The accumulation patterns obtained in this study were in the decreasing order of Zn > Cu > Pb > Cd.

### 3.1. Variations in Ability of Organs Accumulation of Metal

Findings showed that the concentrations of the metals in the organs were in the order of liver > gills > flesh. The essential metals Zn and Cu and nonessential metal Pb indicated higher bioaccumulation in the liver. Cd recorded higher concentration in gills.

The bioaccumulation of metals in liver may be linked to its function of metabolism [4], that is, chemical processes that occur within a living organism in order to maintain life. High levels of Zn and Cu in the liver also are connected to natural protein binding such as Metallothioneins [18]. Liver serves as store for metals, redistribution, and detoxification [19]. This is the yardstick for which liver organs are regarded as an indicator of water pollution than any organ in fishes [1]. Similar results of high Zn and Cu in liver were recorded by other researchers [4, 20, 21].

Some species of the fish indicated bioaccumulation of Cd in gills. Gills are pathways of metal ion exchange from water [22], because gills have very wide surface area that fastens diffusion of metals rapidly [23]. Hence, it is suggested that metals bioaccumulated in gills are basically concentrated from water. This is in agreement with the results of Moore and Ramamorthy [24]. Similar results of high Cd accumulations in gills were reported by Kargin [25], Avenant-Oldewage and Marx [26], Abu-Hilal and Ismail [27], and Qadir and Malik [22].

In this study, fish were collected covering different feeding habitat as indicated in Table 1. The results showed that fish exhibited wide range of variations in interspecific metal concentration in all organs. Several studies indicated high metal concentration to feeding habitat of the fish. Khalid [28] argued that* Sirivutas* being an herbivore thus bioaccumulate higher metal concentration in their flesh than the carnivore* Sargus.* This suggestion is in an agreement with the current study as* Heterotis* (herbivore) recorded higher concentration of metals in flesh than* Clarias* (Carnivore). Abdallah [29] recorded high concentration of Pb and Zn in the flesh of* sardinella aurita* collected from EL-Mex Bay.

These findings are linked to feeding of the fish on phytoplankton because it is the probable biota compartment for Cu and Zn concentration [29–31].

However, a large size of fish of* Heterotis niloticus* showed higher concentrations of Cd in liver than the other studied fish. This finding can be linked to the age of the fish because according to Khalid [28], Cd is not easy to be excreted once it is accumulated in the liver. This large fish (length 36.5–58.5 cm, weight 403.6–1370 g) likely accumulated high Cd throughout its long life. This is in agreement with suggestion of Eisler [20] that Cd in liver is linked to the age of the fish. Furthermore, Ploetz et al. [32] suggested that Cd accumulations in the liver of King mackerel* (Scomberomorus cavalla)* increased with increasing fork length. Kojadinovic et al. [33] recorded Cd concentration in liver of swordfish* (Xiphias gladius)* up to 46.9 mg/kg wet wt.

Results of this study are in agreement with report of Abdallah [29] and Nweeze et al. [30] in which pelagic fish (omnivorous/herbivore) recorded higher metals concentrations than the benthic fish (carnivore) (Table 2). Although fish are mostly migratory and seldom settle in one location, metal accumulation in fish organs gives evidences of exposure to polluted aquatic environment [22] and could be used to evaluate the health condition of the environment from which they were collected. In this study, spatial distribution of metals in the organs of the studied fish species from Lake Geriyo is mainly due to anthropogenic input of metals as it is not near any industrial location.

The consumption of these metals in excess could impact health hazards to human. Zinc might cause nausea, vomiting, diarrhea, metallic taste, kidney, and stomach damage. Zinc is safe when taken in not larger than the recommended amount by Amiard et al. [19]. Acute symptoms of copper contamination by ingestion include vomiting, hematemesis (vomiting blood), and gastrointestinal distress [34]. Young children are vulnerable to toxic effects of lead and can suffer profound and permanent adverse health effects, particularly the development of brain and nervous system. Lead also causes long-term harm in adults, including increased risks of high blood pressure, kidney damage, and neurological effects [5]. Ingestion of any significant amount of cadmium causes immediate poisoning and damage to liver and kidneys. Compounds containing cadmium are also carcinogenic (El-Moselhy et al. 2014).

### 3.2. Health Risk Assessment for Fish Consumption

To assess public health risk of Lake Geriyo fish consumption, metal concentrations in flesh of the fishes in this study were compared with the Maximum Permissible Limits (MPL) for human consumption as set by various organizations (Table 3).

The concentrations of metals in the studied fish species from Lake Geriyo fell below the MPL for human consumption recommended by FAO [13]; FAO/WHO [14]; WHO [15], and England [16] with few exceptions (Table 3). The essential metals Zn and Cu were clearly below the permissible limit (PML), for human consumption. Similarly the nonessential metal Pb was below PML recommended by FAO [13]; FAO/WHO [14]; WHO [15], and England [16] only for fish species of* Clarias* and* Tilapia* in the dry season, while* Clarias* and* Tilapia* in wet season and* Heterotis* in both seasons were above the standard. Cd nonessential metal was below the recommended limit of PML. The fish species, namely,* Clarias* and* Tilapia* in wet season and* Heterotis* in both seasons, of Lake Geriyo were found to be unsafe for consumption. They were contaminated by Pb and a threat to public health. This could be likely due to anthropogenic sources as the lake is not closed to industrial area.

## 4. Conclusions

The result showed that metal accumulation varied depending on species-specific factors; others are feeding behavior, fish size, and age. Metal concentrations were higher in omnivorous/herbivore fish such as* Heterotis niloticus* and lower in carnivore fish such as* Clarias anguillaris*. Variations in metal concentrations were recorded in the internal organs of the studied fish species. Metal accumulations were higher in the liver followed by gills and flesh. Health risk analysis of heavy metals in the edible parts of the fish indicated safe levels for human consumption and concentration in the flesh are generally accepted by the international legislation limit for essential metals (Zn and Cu) and nonessential metal (Cd). However, Pb in* Clarias* and* Tilapia *fish species during the wet season and* Heterotis* in both seasons exceeded MPL, hence unsafe for consumption, and therefore they pose a threat to public health.

## Figures and Tables

**Table 1:** Fish species ecological characteristics.

Scientific name	English name	Feeding habit	Biotype complex	Number of samples	Length (cm)		Weight (g)	
					D	W	D	W
*Clarias anguillaris *	Cat fish	Carnivore (fish & invertebrate)	Benthnic	5	27.80–41.00	30.5–49.2	150–458	186.5–712.8
*Heterotis niloticus*	African bony tongue	Omnivorous/herbivore	Pelagic	5	17.40–20.20	36.5–58.5	53.6–86.1	403.6–1370
*Tilapia zilli*	Mango fish	Omnivorous/herbivore	Pelagic	5	14.50–17.90	19.3–23.5	36.8–101.5	125.2–180.4

D = dry season; W = wet season.

**Table 2:** Seasonal variations in heavy metals mean concentrations (mg/kg) within the flesh, liver, and gills of* Clarias, Tilapia,* and *Heterotis* from Lake Geriyo.

Heavy metals	Season	*Clarias* fish specie			*Tilapia* fish specie			*Heterotis* fish specie		
		Flesh	Liver	Gills	Flesh	Liver	Gills	Flesh	Liver	Gills
Zn	Dry	5.35 ± 1.19^d^	22.4 ± 1.19^c^	16.63 ± 3.41^c^	4.48 ± 0.46^d^	97.28 ± 35.72^ab^	10.13 ± 2.76	10.18 ± 2.41^c^	148.08 ± 19.47^a^	10.17 ± 3.88^c^
	Wet	4.17 ± 0.39^c^	65.04 ± 19.39^a^	10.45 ± 0.60^e^	5.06 ± 0.38^c^	52.87 ± 14.40^c^	13.34 ± 1.4^e^	6.61 ± 0.89^c^	125 ± 10.68^a^	25.94 ± 5.56^d^
Pb	Dry	0.23 ± 0.22^b^	3.5 ± 0.00^ab^	3.42 ± 1.97^a^	0.01 ± 0.00^b^	0.5 ± 0.0^b^	5.39 ± 3.15^a^	3.51 ± 1.30^a^	4.5 ± 0.00^b^	3.30 ± 2.01^a^
	Wet	8.44 ± 2.54^a^	1.22 ± 0.76^b^	4.00 ± 2.10^ab^	3.78 ± 1.36^b^	5.55 ± 1.77^a^	4.79 ± 2.58^ab^	7.12 ± 2.62^a^	8.99 ± 3.78^a^	2.17 ± 0.90^b^
Cd	Dry	0.54 ± 0.14^b^	0.11 ± 0.01^d^	0.53 ± 0.19^b^	0.30 ± 0.15^c^	0.17 ± 0.07^d^	0.42 ± 0.20^b^	0.35 ± 0.09^bc^	0.30 ± 0.00^d^	0.79 ± 0.25^a^
	Wet	0.37 ± 0.25^b^	0.29 ± 0.10^b^	0.26 ± 0.07^b^	0.33 ± 0.16^b^	0.44 ± 0.16^ab^	0.61 ± 0.14^a^	0.39 ± 0.11^b^	0.51 ± 0.20^a^	0.48 ± 0.23^ab^
Cu	Dry	0.5 ± 0.00^e^	29.87 ± 9.28^a^	0.15 ± 0.00^c^	0.15 ± 0.00^c^	31.15 ± 3.40^a^	0.15 ± 0.00^c^	0.15 ± 0.00^c^	20.4 ± 4.19^b^	0.15 ± 0.00^c^
	Wet	2.02 ± 1.87^bc^	22.6 ± 7.85^a^	0.31 ± 0.16^c^	1.27 ± 1.12^c^	18.08 ± 6.54^a^	2.62 ± 1.65^bc^	4.42 ± 1.89^b^	4.69 ± 2.05^b^	1.41 ± 0.78^c^

Data on the same row with different superscripts are significantly difference (*P* < 0.05).

**Table 3:** Heavy metals in flesh (mg/kg, ww) of fish from Lake Geriyo and Maximum Permissible Limits (MPL) international standard.

Heavy metals	Concentrations of metals (mg/kg, ww) in different species						MPL			
	*Claris*		*Tilapia*		*Heterotis*		FAO [13]	FAO/WHO [14]	WHO [15]	England [16]
	D	W	D	W	D	W				
Zn	5.35	4.17	4.48	5.06	10.18	6.61	30	40	100	50
Pb	0.23	8.44	0.01	3.78	3.51	7.12	0.5	0.5	2	2
Cd	0.54	0.37	0.30	0.33	0.35	0.39	0.05	0.5	1	2
Cu	0.15	2.02	0.15	1.27	0.15	4.42	30	30	30	20

D = dry season; W = wet season.

## Data Availability

The data used to support the findings of this study are available from the corresponding author upon request.

## References

[1] El-Moselhy K. M. (2000). Accumulation of copper, cadmium and lead in some fish from the Guif of suez.

[2] Kris-Etherton P. M., Harris W. S., Appel L. J. (2002). Fish consumption, fish oil, omega-3 fatty acids, and cardiovascular disease.

[3] Yilmaz F., Özdemir N., Demirak A., Tuna A. L. (2007). Heavy metal levels in two fish species *Leuciscus cephalus* and *Lepomis gibbosus*.

[4] Zhao S., Feng C., Quan W., Chen X., Niu J., Shen Z. (2012). Role of living environments in the accumulation characteristics of heavy metals in fishes and crabs in the Yangtze River Estuary, China.

[5] Voegborlo R. B., Atta A., Agorku E. S. (2012). Total mercury distribution in different tissues of six species of freshwater fish from the Kpong hydroelectric reservoir in Ghana.

[6] Castro-González M. I., Méndez-Armenta M. (2008). Heavy metals: implications associated to fish consumption.

[7] Al-Busaidi M., Yesudhason P., Al-Mughairi S. (2011). Toxic metals in commercial marine fish in Oman with reference to national and international standards.

[8] Rahman M. S., Molla A. H., Saha N., Rahman A. (2012). Study on heavy metals levels and its risk assessment in some edible fishes from Bangshi River, Savar, Dhaka, Bangladesh.

[9] Meche A., Martins M. C., Lofrano B. E., Hardaway C. J., Merchant M., Verdade L. (2010). Determination of heavy metals by inductively coupled plasma-optical emission spectrometry in fish from the Piracicaba River in Southern Brazil.

[10] Mustafa C., Guluzar A. (2003). The relationships between heavy metal (Cd, Cr, Cu, Fe, Pb, Zn) levels and the size of six Mediterranean fish species.

[11] Ekundayo T. M., Sogbesan O. A., Haruna A. B. (2014). Study of fish exploitation pattern of lake Gerio, Yola, Adamawa State, Nigeria.

[12] Adebayo A. A., Tukur A. L. (1999).

[13] Food and Agriculture Organization (1983). Compilation of legal limits for hazardous substances in fish and fishery production.

[14] FAO/WHO (1989). WHO technical report series No 505, Evaluation of certain food additives and the contaminants, mercury, lead and cadmium for environment monitory report No 52 center for environment.

[15] WHO World Health Organization (1995). Heavy metals environmental aspects.

[16] England Aquatic environment monitoring report no. 52.

[17] Bernhard M. (1976). Manual of methods in aquatic environment research , parts 3. Sampling and analyses of biological material.

[18] Gorar F. K., Keser R., Akiel N., Dizman S. (2012). Radioactivity and heavy metal concentrations of some commercial fish.

[19] Amiard J., Amiardtriquet C., Barka S., Pellerin J., Rainbow P. (2006). Metallothioneins in aquatic invertebrates: Their role in metal detoxification and their use as biomarkers.

[20] Eisler R. (2010).

[21] Dural M., Göksu M. Z. L., Özak A. A. (2007). Investigation of heavy metal levels in economically important fish species captured from the Tuzla lagoon.

[22] Qadir A., Malik R. N. (2011). Heavy Metals in Eight Edible Fish Species from Two Polluted Tributaries (Aik and Palkhu) of the River Chenab, Pakistan.

[23] Dhaneesh K. V., Gopi M., Ganeshamurthy R., Kumar T. T., Balasubramanian T. (2012). Bio-accumulation of metals on reef associated organisms of Lakshadweep Archipelago.

[24] Moore J. W., Ramamorthy (1989).

[25] Kargin F. (1998). Metal Concentrations in Tissues of the Freshwater Fish Capoeta barroisi from the Seyhan River (Turkey).

[26] Avenant-Oldewage A., Marx H. M. (2000). Bioaccumulation of chromium, copper and iron in the organs and tissues of *Clarias gariepinus* in the Olifants River, Kruger National Park.

[27] Abu-Hilal A. H., Ismail N. S. (2000). Heavy metals in eleven common species of fish from the Gulf of Agaba.

[28] Khalid A. (2004). Seasonal determination of soil heavy metals on muscles tissues of siganus revaltus and sargus sargus fish from El-mex bay and Eastern Harbor, Alexandra, Egypt.

[29] Abdallah M. A. M. (2008). Trace element levels in some commercially valuable fish species from coastal waters of Mediterranean Sea, Egypt.

[30] Nweeze N. O., Mahmood L. B., Aisha U. I. (2014). Lithological Studies and Aigal diversity, the useful fool for assessment of fish pond water quality.

[31] EPA (1972).

[32] Ploetz D. M., Fitts B. E., Rice T. M. (2007). Differential accumulation of heavy metals in muscle and liver of a marine fish, (king mackerel, *Scomberomorus cavalla* cuvier) from the Northern Gulf of Mexico, USA.

[33] Kojadinovic J., Potier M., Le Corre M., Cosson R. P., Bustamante P. (2007). Bioaccumulation of trace elements in pelagic fish from the Western Indian Ocean.

[34] Arvind K. (2002).

